# Associations Among Personality Traits, Stress Experiences, and Physical and Cognitive Outcomes in Older Adults

**DOI:** 10.1093/geroni/igab046.034

**Published:** 2021-12-17

**Authors:** Eileen Graham, David Almeida

## Abstract

Understanding between and within person variability in personality traits, and the processes of general and perceived stress are essential to understanding how to optimize cognitive health in older adults. It is well known that there is large variation in cognitive change: the pace and direction of change differs greatly across individuals. Personality traits and stress experiences are key factors that may account for some of these individual differences. The goal of our symposium is to present novel research in this area and discuss the implications for understanding personality, stress, and cognitive decline. First, Ferguson and colleagues will present a novel approach to assessing daily variability in personality. Their results demonstrate that daily personality assessments are able to capture within-person variability in personality, which could potentially help predict health trajectories in later adulthood. This is an important step in the study of change processes. Second, Luo and colleagues will present the factor structure of general and perceived stress, and show the predictive utility of these factors on physical and cognitive health outcomes. Third, Lawson and colleagues will discuss the extent to which personality is associated with cognitive function in a large sample of Mexican-origin adults. Fourth, Graham and colleagues will present results from a coordinated analysis that addressed associations among personality traits and cognitive decline both pre- and post- dementia diagnosis. Discussant David Almeida will contextualize these new findings and propose next steps.

